# Visual detection and characterization of chronic myocardial infarctions in patients using native T_1_ maps at 3T

**DOI:** 10.1186/1532-429X-17-S1-O74

**Published:** 2015-02-03

**Authors:** Avinash Kali, Eui-Young Choi, Behzad Sharif, Young Jin Kim, Xiaoming Bi, Bruce S Spottiswoode, Ivan Cokic, Hsin-Jung Yang, Mourad Tighiouart, Debiao Li, Daniel S Berman, Byoung Wook Choi, Hyuk-Jae Chang, Rohan Dharmakumar

**Affiliations:** 1 Cedars-Sinai Medical Center, Los Angeles, CA, USA; 2 Yonsei University College of Medicine, Seoul, Korea (the Republic of; 3 Siemens Healthcare, Los Angeles, CA, USA

## Background

Late Gadolinium Enhancement (LGE) Cardiovascular Magnetic Resonance (CMR) is routinely used for characterizing chronic myocardial infarctions (cMIs), but it is contraindicated in patients with end-stage chronic kidney disease. We investigated whether native T_1_ mapping at 3T can be used to detect and characterize cMIs in patients with prior STEMI and NSTEMI.

## Methods

Breath-held 2D native T_1_ maps (8 TIs with 2 Look-Locker cycles of 3+5 images; minimum TI = 120ms; TI increment = 80 ms; flip angle = 35°; bandwidth = 1085 Hz/pixel; voxel size = 1.5x1.5x8mm^3^) and LGE images (IR-prepared FLASH; optimal TI to null remote myocardium; TR/TE = 6.54/3.27ms; flip angle = 20°; bandwidth = 460 Hz/pixel; voxel size = 1.2x1.2x8mm^3^) were acquired in patients with prior STEMI (n=15) and NSTEMI (n=17) at 3T at a median of 13.6 years after acute MI. cMI location, size and transmurality were determined using Mean+5SD criterion relative to remote myocardium. Visual detection of cMI territories on LGE images and T_1_ maps were assessed by two independent reviewers.

## Results

**Figure 1:**
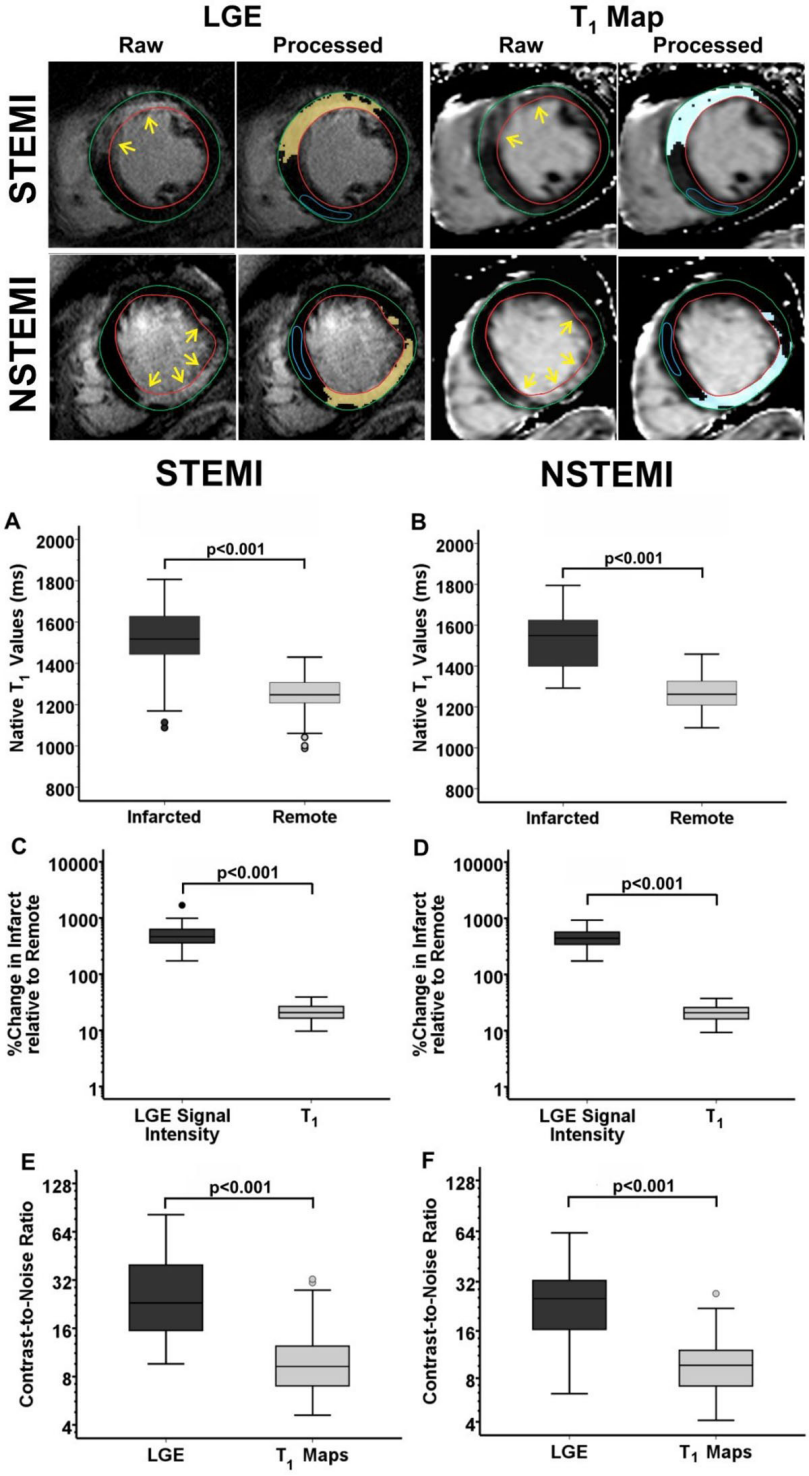
Representative native T1 maps and LGE images from a patient with prior STEMI (infarct age = 18.5 years) and NSTEMI (infarct age = 25.6 years) are shown. Significant T1 elevations could be observed within the infarcted regions detected on LGE images (arrows). Good visual agreement was observed between the two techniques in terms of the location and spatial extent of the infarct. Median native T1 of the infarct was significantly elevated in both STEMI and NSTEMI patients. Percentage change in LGE signal intensity and infarct-to-remote CNR was significantly in LGE images were significantly higher than that of T1 maps.

**Figure 2:**
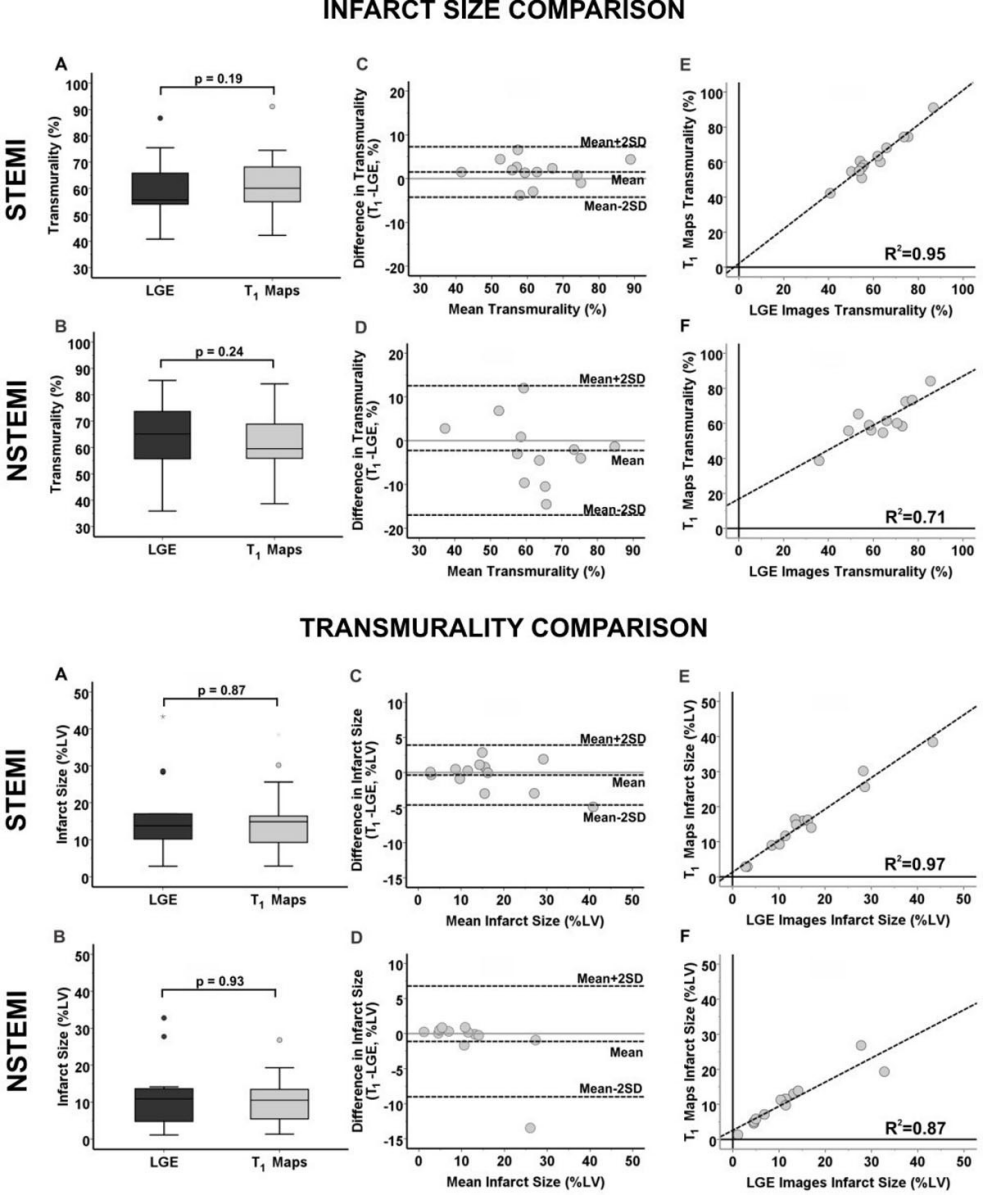
Native T1 maps and LGE images were not different for measuring chronic infarct size and transmurality in both STEMI and NSTEMI patients. Bland-Altman and linear regression analyses further showed good agreement for measuring infarct size and transmurality.

Representative native T_1_ maps and LGE images from two patients, one with prior STEMI, and one with prior NSTEMI are shown in Fig. 1. Relative to remote myocardium, median T_1_ of the cMI was 271ms higher in STEMI patients (Infarct: 1517ms; Remote: 1247ms; p<0.001; Fig. 1), and 229ms higher in NSTEMI patients (Infarct: 1549ms, Remote: 1262ms; p<0.001; Fig. 1). Median percentage change in LGE signal intensity (LGE-SI) of the cMI relative to remote myocardium was significantly higher than that of percentage change in T_1_ in both STEMI (LGE: 465%, T_1_: 21%; p<0.001) and NSTEMI (LGE: 441%, T_1_: 20%; p<0.001) patients. Median CNR of LGE images was also 2.5-fold higher relative to that of T_1_ maps in both STEMI (LGE: 23.1; T_1_: 9.2; p<0.001) and NSTEMI (LGE: 25.3; T_1_: 9.7; p<0.001) patients. LGE images and native T_1_ maps were not different for measuring cMI size (STEMI - LGE: 13.8%; T_1_: 14.9%; p=0.87; NSTEMI - LGE: 10.9%; T_1_: 10.5%; p=0.93; Fig. 2) and transmurality (STEMI - LGE: 55.6%; T_1_: 60.1%; p=0.19; NSTEMI - LGE: 64.3%; T1: 60.9%; p=0.24). Statistical analyses showed good agreement between LGE images and T_1_ maps for measuring cMI size (STEMI: bias=-0.4±2.1%; R^2^=0.97; NSTEMI: bias=-1.1±3.9%; R^2^=0.87) and transmurality (STEMI: bias=1.5±2.9%; R^2^=0.99; NSTEMI: bias=-2.2±7.4%; R^2^=0.71). Sensitivity and specificity of native T_1_ maps for detecting cMIs based on threshold criterion were 93% and 97% respectively (STEMI); and 93% and 92% respectively (NSTEMI). Sensitivity and specificity of native T_1_ maps for visual detection of cMI were: 61% and 85% (STEMI); and 67% and 90% (NSTEMI).

## Conclusions

Native T_1_ maps can reliably detect and characterize cMIs in STEMI and NSTEMI patients when the location of remote myocardium is known. Further increase in image contrast may be necessary to improve visual detection sensitivity of chronic MI territories to the levels observed with LGE.

## Funding

National Heart, Lung, and Blood Institute (RO1 HL091989) and American Heart Association Pre-Doctoral Fellowship (13PRE17210049).

